# Editorial for the Special Issue on Micro Air Vehicles

**DOI:** 10.3390/mi14040721

**Published:** 2023-03-24

**Authors:** Syed Agha Hassnain Mohsan, Muhammad Asghar Khan, Mumtaz Karatas

**Affiliations:** 1 Optical Communication Laboratory, Ocean College, Zhejiang University, Zheda Road 1, Zhoushan 316021, China; 2 Department of Electrical Engineering, Hamdard Institute of Engineering & Technology, Islamabad 44000, Pakistan; 3 Department of Industrial Engineering, Turkish Naval Academy, National Defense University, 34940 Istanbul, Turkey

Recently, Micro Air Vehicles (MAVs) have been receiving a significant amount of attention from research organizations and businesses worldwide due to their unique characteristics, including high mobility, three-dimensional (3D) movement, and ease of deployment. These flying machines have proven their worth in last-minute package delivery during rush hours and base searches in inaccessible regions of battlefields, which humans cannot reach or effectively function in. MAVs can significantly lower the risk to human life, enhance system efficiency, and shorten the time of operations compared to conventional methods. MAV capabilities typically range from surveillance MAVs with fixed wings to modern MAVs capable of hovering, navigation, carrying many sensors, and accomplishing missions up to several kilometers in distance. Aside from these appealing benefits, MAVs encounter certain challenges such as limited energy, low processing power, security breaches, and so on.

In an effort to disseminate the current advancements in the specialized field of MAVs based on theory and experiments in the areas of novel designs for MAVs, innovative strategies for aerial manipulation, localization, control mechanisms for the guidance and navigation of single and multi-MAVs, security, trajectory planning, and tracking, a Special Issue of *Micromachines* has been dedicated to “Micro Air Vehicles”.

This Special Issue presents eight articles covering most of the current topics of research in MAVs, from charging techniques to assisting in the COVID-19 pandemic, and from offering lightweight security schemes to efficient and secure WiFi signal boosters.

Despite their growing popularity in military and civilian applications, charging MAVs remains one of the most difficult and time-consuming tasks. Mohsan et al. [1] elaborated on current WPT approaches and showed that they could considerably enhance MAVs’ autonomous operations, including the technical elements of WPT for MAVs. MAVs or drones can help combat pandemic viruses such as dengue and COVID. Ali et al. [2] proposed a novel privacy-preserved IoMT approach to manage dengue virus outbreaks by monitoring infected patients based on the bedding location inferred from call data record analysis (CDRA). Geographic information system mapping can locate a patient’s infected site after identifying their bedding. Mohsan et al. [3] addressed a number of drone applications for the minimization of the danger of COVID-19, including their use in surveillance, inspection, message broadcasting through QR codes and loudspeakers, medical supply delivery, disinfectant spraying, patient screening, and patient identification.

MAVs are vulnerable to a wide variety of cyber-physical attacks because they often communicate with one another across unencrypted wireless channels. Ullah et al. [4] proposed a lightweight and highly secure conditional privacy-preserving generalized ring signcryption scheme for MAVs using an identity-based cryptosystem. The proposed scheme enables encryption and digital signature both simultaneously and independently and guarantees anonymity, spontaneity, flexibility, and equal membership. According to practical studies and analyses of UAVs’ localization and clustering, the method presented by Amran et al. [5] can increase the amplitude of wireless signals in distant places to safeguard the information transmitted by UAVs using blockchain technology as a decentralized database.

Multi-UAV integrated aerial platforms (IAPs) provide payload and fault-tolerance advantages compared to single UAVs, making them ideal candidates for platforms with integrated-response, observation, and striking capabilities. Shi et al. [6] proposed an IAP configuration design with three sub-UAVs, investigated the geometric control of the integrated multi-UAV system, and developed an IAP prototype composed of software and hardware systems, offering a design and control foundation for building future multi-UAV IAPs. In addition, Corregidor-Castro et al. [7] aimed to determine the advantages and disadvantages of the use of UAVs as monitoring tools to census colonies of two species of breeding seabirds, the Herring Gull (Larus argentatus) and the Lesser Black-backed Gull (Larus fuscus). Finally, Ji et al. [8] provided theoretical references and technical support for the verification of the feasibility of the Trichogramma delivery system.
